# Saponin-Induced Shifts in the Rumen Microbiome and Metabolome of Young Cattle

**DOI:** 10.3389/fmicb.2019.00356

**Published:** 2019-02-28

**Authors:** Bing Wang, Man Peng Ma, Qi Yu Diao, Yan Tu

**Affiliations:** ^1^Feed Research Institute, Chinese Academy of Agricultural Sciences, Beijing, China; ^2^College of Animal Science and Technology, China Agricultural University, Beijing, China

**Keywords:** 16S rRNA gene, cattle, metabolomics, microbiome, rumen, saponins, UHPLC-QTOF-MS

## Abstract

The aim of this study was to explore the effects of saponins on the rumen microbiota and the ruminal metabolome. Alfalfa hay (AH) and soybean hulls (SH) were used as fiber sources for the control diets. The AH and SH diets were supplemented with tea saponins resulting in two additional diets named AHS and SHS, respectively. These 4 diets were fed to 24 young male Holstein cattle (*n* = 6 per diet). After 28 days of feeding, the rumen fluid from these cattle was collected using an oral stomach tube. Illumina MiSeq sequencing and ultrahigh-performance liquid chromatography coupled to quadrupole time-of-flight mass spectrometry (UHPLC-QTOF/MS) were used to investigate the changes in the ruminal microbes and their metabolites. The relative abundance of *Prevotellaceae_YAB2003* increased, while *Ruminococcaceae_NK4A214* and *Lachnospiraceae_NK3A20* decreased in SHS and AHS compared to SH and AHS, respectively. Feeding SHS resulted in higher ruminal concentrations of squalene, lanosterol, 3-phenylpropanoic acid, and citrulline compared to SH. The different microbial genes predicted by Tax4Fun were involved in amino sugar and nucleotide sugar metabolism. The pathways of arginine and proline metabolism, purine metabolism, and pyrimidine metabolism were enriched by different metabolites. Moreover, in the SH group, a positive correlation was observed between *Prevotella_1* (*Bacteroidetes*), *Prevotellaceae_YAB2003* (*Bacteroidetes*), and *Christensenellaceae_R.7* (*Firmicutes*), and the metabolites, including citrulline, lanosterol, and squalene. The increased abundances of *Prevotella_1*, *Ruminococcaceae_UCG.002*, and *Prevotellaceae_YAB2003* might result in increased fiber digestion and nutrient utilization but nutrient digestion was not measured in the current study. In summary, saponins have the ability to modulate the ruminal microbial community and ruminal metabolites and thus affect the rumen environment. However, the response seems to be dependent on the composition of the basal diet. This study provides a comprehensive overview of the microbial and biochemical changes in the rumen of cattle fed saponins.

## Introduction

In recent years, plant-derived bioactive compounds, such as saponins, have been discussed as potential alternatives to conventional antibiotics for use as growth promoters in ruminant production (Benchaar et al., 2008; Kamra et al., 2008). Tea saponins, which primarily contain triterpenoid saponins, have been reported to act as antimicrobials, modifying rumen fermentation by decreasing rumen protozoa numbers and reducing losses of enteric methane (Wang et al., 2012). Depending on the source and dosage, tea saponins also have the potential to improve the milk production performance of lactating dairy cows (Wang et al., 2017).

The microbial community of the rumen is complex and consists of bacteria, protozoa, archaea, and fungi. It has been established that the composition and activity of the rumen microbiota have a profound impact on the performance, health, and immune system of the host (Jami et al., 2014; Huws et al., 2018). A better understanding of the composition of rumen microbial communities and the interaction between the microbiome and the metabolome is crucial to understand the impact of plant-derived feed additives such as saponins on rumen fermentation. For example, saponins may not only have adverse effects on rumen protozoa (Wang et al., 2012) but may also affect specific bacteria and fungi, which may alter rumen metabolism (Guo et al., 2008; Wang et al., 2012). To the best of our knowledge, no previous studies investigated the effects of saponins on rumen fermentation using an integrative metabolomic and microbiomic approach. In addition, it has been reported that the effect of tannins on lamb production is dietary protein level-dependent (Śliwiński et al., 2002). The effects of saponins on *in vitro* rumen fermentation and the microbial composition are influenced by diet composition (Patra and Yu, 2015). Growing cattle need fiber for rumen development, and soybean hulls and alfalfa hay are two fiber sources that are widely used in cattle production (Richards et al., 2006; Jahani-Moghadam et al., 2015). In this study, we hypothesized that the effects of tea saponins on the rumen microbiota might be influenced by the dietary fiber source and that an interaction between the rumen microbiota and rumen metabolites exists, which would demonstrate a regulatory role of saponins on rumen metabolism. The aim of this study was to elucidate the effects of saponins on the dynamic changes in the ruminal metabolome, microbiome, and metabolic network in young growing cattle.

## Materials and Methods

### Animals, Diets, and Experimental Design

The young cattle selected for this study were provided by a commercial dairy farm (Henan Agriculture University Test Base, Xuchang, China). The experimental procedures were approved by the Animal Ethics Committee of the Chinese Academy of Agricultural Sciences (Beijing, China). Saponins (56–60% purity of triterpenoid saponins) in powder form that were extracted from camellia seed (*Camellia oleifera* Abel) were provided by the Zhejiang Orient Tea Industry (Shaoxing, China). Twenty-four clinically healthy Chinese Holstein bull calves (age = 150 ± 3 days; body weight = 150 ± 3.9 kg) were randomly assigned to one of four treatments (*n* = 6 per treatment): alfalfa hay (AH) and soybean hull diets (SH; Supplementary Table S1), a diet with saponins supplemented at 9 g/calf per day and AH (AHS), and a diet with saponins supplemented at 9 g/calf per day and SH (SHS). The effects of saponins on rumen fermentation, the metabolome and the microbiome were examined by comparing AHS to AH and SHS to SH, respectively. The supplementation rate of saponins was chosen based on a previous study of our laboratory that showed increased milk yield and oxidative resistance when saponins were fed at 30 g/d to lactating dairy cows (body weight = approximately 500 kg; Wang et al., 2017). The supplementation rate was adjusted according to the lower body weight of the young cattle. The saponins were supplemented after 28 days of adaptation to AH and SH. The saponin treatment lasted for 28 days with the first 7 days for adaptation. Clean, fresh water and the total mixed ration were provided *ad libitum*. The cattle were fed twice daily at 0800 and 1600 h, and they were housed in a naturally ventilated barn and kept in individual pens (1.6 m × 3.6 m) that were bedded with wood shavings (Makoto Farming Equipment Co., Ltd.,Beijing, China).

### Sample Collection and Measurements

The collection of ruminal fluid was performed 3 h after the morning feeding on day 28 after the supplementation of saponins. Rumen fluid (100 mL) was collected using an oral stomach tube according to Shen et al. (2012), and the initial 150 mL was discarded to avoid saliva contamination. The pH was measured immediately. The sample was retained to measure rumen volatile fatty acids (VFAs) and rumen bacteria. The samples were immediately flash-frozen in liquid nitrogen and stored at −80°C until further analysis. One subsample was used for DNA extraction and subsequent microbial quantification, and another subsample was used for the metabolomic analysis. The remaining sample was used to measure VFAs according to Hu et al. (2005). Briefly, the VFA were analyzed by gas chromatography (GC-8A; Shimadzu Corp., Kyoto, Japan) after ortho-phosphoric acid (25% w/v) was added to filtered rumen fluid and centrifuged (17,000 *g* for 10 min at 4°C).

### DNA Extraction and Sequencing

Microbial DNA was extracted from ruminal fluid samples using the E.Z.N.A. stool DNA kit (Omega Bio-tek, Norcross, GA, United States) according to the manufacturer’s protocols. The 16S rDNA V3–V4 region of the eukaryotic ribosomal RNA gene was amplified by PCR (95°C for 2 min, followed by 27 cycles at 98°C for 10 s, 62°C for 30 s, and 68°C for 30 s and a final extension at 68°C for 10 min) using primers 341F: CCTACGGGNGGCWGCAG and 806R: GGACTACHVGGGTATCTAAT, where the barcode is an eight-base sequence that is unique to each sample. PCRs were performed in a triplicate 50 μL mixture containing 5 μL of 10 × KOD buffer, 5 μL of 2.5 mM dNTPs, 1.5 μL of each primer (5 μM), 1 μL of KOD polymerase, and 100 ng of template DNA.

Amplicons were extracted from 2% agarose gels and purified using the AxyPrep DNA Gel Extraction Kit (Axygen Biosciences, Union City, CA, United States) according to the manufacturer’s instructions and quantified using QuantiFluor-ST (Promega, United States). Purified amplicons were pooled in equimolar ratios and paired-end sequenced (2 × 250) on an Illumina platform by Hiseq2500 PE250 according to the standard protocols. The raw reads were deposited into the NCBI Sequence Read Archive (SRA^1^) database (Accession Number: SRP151436).

### Bioinformatic Analysis

Raw data containing adapters or low-quality reads would affect the following assembly and analysis. Therefore, the raw reads were filtered. Reads containing more than 10%, unknown nucleotides and reads containing less than 80% of bases with a *Q*-value of >20 were removed. Paired-end clean reads were merged as raw tags using FLSAH (v 1.2.11) with a minimum overlap of 10 bp and mismatch error rates of 2% (Magoč and Salzberg, 2011). Noisy sequences of raw tags were filtered by the QIIME (V1.9.1; Caporaso et al., 2010) pipeline under specific filtering conditions (Bokulich et al., 2013) to obtain clean tags. To perform a reference-based check for chimera, the clean tags were compared against the reference database^2^ using UCHIME^3^. All chimeric tags were removed to obtain effective tags for further analyses.

The effective tags were clustered into operational taxonomic units (OTUs) of ≥ 97% similarity using the UPARSE pipeline (Edgar, 2013). The tag sequence with the highest abundance was selected as the representative sequence within each cluster. Between groups, a Venn analysis was performed in R (Version 3.2.4) to identify unique and common OTUs. The representative sequences were classified as organisms by a naive Bayesian model using an RDP classifier (Version 2.2; Wang et al., 2007) that was based on the SILVA (Pruesse et al., 2007) database^4^. The abundance of each taxonomy and phylogenetic tree was constructed in Perl script and visualized using SVG. Metastats was utilized to select and demonstrate the differentially abundant taxonomies between the groups. Chao1, Simpson and all other alpha diversity indices were calculated in QIIME. The OTU rarefaction curve and rank abundance curves were plotted in QIIME. The statistics of the between-group alpha index comparisons were calculated by Welch’s *t*-test and a Wilcoxon rank test in R. To compare the alpha indices among groups, Tukey’s HSD test and a Kruskal–Wallis *H* test were performed in R (Version 3.2.4). A weighted and unweighted UniFrac distance matrix was generated by QIIME. Multivariate statistical analyses, including a principal coordinates analysis (PCoA) of unweighted UniFrac distances, were calculated and plotted in R (Version 3.2.4). The functional group (guild) of the OTUs was inferred using Tax4Fun (v1.0; Asshauer et al., 2015).

### Metabolite Extraction

After thawing at room temperature, the ruminal fluid sample (100 mg) was removed and placed in an EP tube, and then 1000 μL of extraction liquid (V methanol: V acetonitrile: V water = 2:2:1, kept at −20°C before extraction) with 20 μL of internal standard was added. The mixture was then homogenized in a ball mill for 4 min at 45 Hz and ultrasound treated for 5 min (cooled). After being homogenized 3 times, the mixture of pretreated rumen fluid was incubated for 1 h at −20°C to precipitate the proteins. The rumen fluid was then centrifuged at 12,000 *g* for 15 min at 4°C. The supernatant (700 μL) was transferred into EP tubes, and the extracts were dried in a vacuum concentrator without heating (rumen temperature). Then, 200 μL of extraction liquid (V acetonitrile: V water = 1:1) was added, and the mixture was vortexed for 30 s, sonicated for 10 min (4°C water bath), and centrifuged (*g* for 15 min at 4°C). The supernatant (60 μL) was transferred into a fresh 2 mL LC/MS glass vial, and 10 μL of each sample was taken and pooled as quality control samples. Then, 60 μL of supernatant was used for the UHPLC-QTOF-MS analysis.

### LC-MS/MS Analysis

The LC-MS/MS analyses were performed using an UHPLC system (1290, Agilent Technologies) with a UPLC BEH Amide column (1.7 μm 2.1 mm × 100 mm, Waters) coupled to Triple TOF 5600 (Q-TOF, AB Sciex). The mobile phase consisted of 25 mmol/L NH_4_OAc and 25 mmol/L NH_4_OH in water (pH = 9.75) (A) and acetonitrile (B) and was used with an elution gradient as follows: 0 min, 95% B; 7 min, 65% B; 9 min, 40% B; 9.1 min, 95% B; and 12 min, 95% B, which was delivered at 0.5 mL min^−1^. The injection volume was 2 μL. The Triple TOF mass spectrometer was used for its ability to acquire MS/MS spectra on an information-dependent basis (IDA) during an LC/MS experiment. In this mode, the acquisition software (Analyst TF 1.7, AB Sciex) continuously evaluates the full-scan survey MS data as it collects and triggers the acquisition of the MS/MS spectra depending on preselected criteria. In each cycle, 12 precursor ions with an intensity greater than 100 were chosen for fragmentation at a collision energy (CE) of 30 V (15 MS/MS events with a product ion accumulation time of 50 ms each). The ESI source conditions were set as follows: ion source gas 1 at 60 Psi, ion source gas 2 at 60 Psi, curtain gas at 35 Psi, source temperature at 650°C, and ion spray voltage floating (ISVF) at 5000 V or −4000 V in positive or negative modes, respectively.

### Metabolomic Data Preprocessing and Multivariate Statistical Analysis

The MS raw data files were converted to the mzXML format using ProteoWizard and processed by the R package XCMS (version 3.2). The preprocessing results generated a data matrix that consisted of the retention time (RT), mass-to-charge ratio (m/z) values, and peak intensity. The CAMERA package of R was used for peak annotation after XCMS data processing. An in-house MS2 database was used to identify the metabolites. The TIC peak retention time and the peak area of the quality control sample all overlapped well, indicating the appropriate stability of the detection procedure. The variations in retention time, mass accuracy, and peak area for selected ions that were present in the quality control samples covering a range of retention times, masses, and intensities were calculated. The retention time shift was less than 0.3 min; the mass accuracy deviation was less than 5 mDa; and the relative SDs (RSD) of peak areas were below 30%. It was concluded that the LC/MS analytical system had appropriate stability and repeatability and that the acquired data were of good enough quality for subsequent assays.

Differential metabolites (DMs) were identified based on pairwise comparisons between AH and AHS and SH and SHS. The peaks were detected, and the metabolites could be identified through the interquartile range denoising method. Missing raw data values were set at half of the minimum value of the detection limit. In addition, an internal standard normalization method was employed in this data analysis. The resultant three-dimensional data involving the peak number, sample name, and normalized peak area were fed to the SIMCA14.1 software package (V14.1, MKS Data Analytics Solutions, Umeå, Sweden) for principal component analysis (PCA) and orthogonal projections to latent structures discriminant analysis (OPLS-DA). The peak intensities were treated as *X* variables, while the sums of the peak intensities were taken as *Y* variables. All the variables were normalized before PCA and OPLS-DA. The principal component analysis showed the distribution of the origin data. To obtain a higher level of group separation and obtain a better understanding of the variables that were responsible for the classification, supervised OPLS-DA was applied. Based on the OPLS-DA, a loading plot was constructed, which showed the contribution of the variables to the differences between the two groups. The loading plot also showed the important variables that were situated far from the origin, but the loading plot was complex because there were many variables. To refine this analysis, the first principal component of variable importance in the projection (VIP) was obtained. The VIP values exceeding 1 were first selected as changed metabolites. In step 2, the remaining variables were then assessed by Student’s *t*-test (*P*-value < 0.05). The fold-change (FC) value of each metabolite was calculated by comparing the mean values of the peak area obtained from any comparison, and the log_2_FC value was used to indicate the specific variable quantity in the comparison. The *Q*-value of each metabolite was computed from the measurements using the fdrtool package in R (Version 3.2.4), which was used to adjust the false discovery rate in the comparisons. The DMs were identified and validated using various databases, including the Kyoto Encyclopedia of Genes and Genomes (KEGG), Human Metabolome Database (HMDB), Bovine Metabolome Database (BMDB), PubChem Compound, Chemical Entities of Biological Interest (ChEBI), Japan Chemical Substance Dictionary Web (NIKKAJI), and Chemical Abstracts Service (CAS).

### Construction of the Metabolic Pathway

In addition, MetaboAnalyst 4.0, which is a web-based tool for the visualization of metabolomics^5^, was utilized to search for the pathways of the DMs (Chong et al., 2018). The analysis used the *Bos taurus* (cow) pathway library, an integrated global test pathway enrichment analysis, and a relative-betweenness centrality pathway topology analysis. All of the matched pathways, according to the *p*-values from the pathway enrichment analysis and pathway impact values from the pathway topology analysis, are shown in the metabolome map. The pathways with both high impact values and *p*-values were regarded as key pathways.

### Correlations Between Microbial Communities and Rumen Metabolites

The different rumen metabolites with a VIP > 1.5 and *P* < 0.05 and different microbial genera (*P* < 0.05 and relative abundance > 0.05% in at least one of the samples) were used for an interactive analysis in R (Version 3.2.4). To explore the functional correlation between the rumen bacterial changes and metabolite perturbations, a Spearman’s rank correlation matrix was generated by calculating the Spearman’s correlation coefficient among the taxa that were affected by the diet type (at the genus level, *P* < 0.05) and candidate ruminal compounds (VIP > 1.5 and *P* < 0.05) in the R program, and only connections with a *P*-value of less than 0.01 and *r* > 0.71 were retained. These correlations were visualized using the Pheatmap^6^ (author, R. Kolde; published date, 2015; version, 1.0.8) package in R and Cytoscape 3.6.1 (Smoot et al., 2011).

### Statistical Analysis

The rumen fermentation characteristics were analyzed as a randomized complete block design using PROC MIXED of SAS (version 9.2, SAS Institute Inc., Cary, NC, United States). Dietary treatment was a fixed effect, and the young growing cattle within each diet were a random effect. The covariance structure with the lowest Akaike information criterion was used. The results were reported as least squares means that were calculated and separated using the PDIFF option in SAS. Significance was declared at *P* < 0.05, and a tendency was declared at 0.05 ≤*P* < 0.10.

## Results

### Rumen Fermentation Characteristics

Rumen pH was similar among the treatments (Table 1). The total VFA concentration had a tendency to be higher in AHS compared to AH (*P* = 0.07). The ruminal acetate proportion was lower (*P* = 0.04) in AHS compared to AH. The ruminal valerate proportion had a tendency to be higher in AHS compared to SH (*P* = 0.08).

**Table 1:** Ruminal pH and volatile fatty acid (VFA) in young growing cattle fed 2 experimental diets based on alfalfa hay (AH) and soybean hull (SH), as well as saponins treated AH (AHS) and saponins treated SH (SHS).

Items	Treatments				Treatments			
	AH	AHS	SEM	*P*-value	SH	SHS	SEM	*P*-value
pH	6.29	6.43	0.127	0.48	5.44	5.41	0.014	0.14
Total VFA, *mM*	29.8	44.5	4.49	0.07	84.9	78.7	3.81	0.31
Molar								
**proportion, %**								
Acetate	66.2	59.7	1.66	0.04	45.6	46.3	1.16	0.69
Propionate	24.1	28.4	1.77	0.15	42.1	40.5	0.94	0.30
Butyrate	8.00	10.2	1.01	0.19	11.0	12.0	1.23	0.60
Valerate	0.28	0.46	0.058	0.08	0.79	0.75	0.099	0.82
Isobutyrate	0.31	0.28	0.058	0.80	ND^a^	ND		
Isovalerate	1.07	0.97	0.158	0.68	0.43	0.41	0.033	0.73

### Saponin-Induced Ruminal Bacteria Changes

**FIGURE 1:**
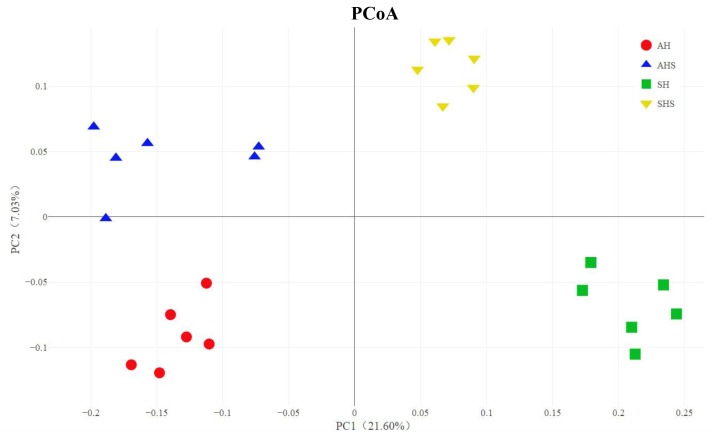
Unweighted Principal coordinates analysis (PCoA) analysis of taxonomical classifications of rumen bacterial communities in the alfalfa hay diet (AH) and saponins treated AH diet (AHS), as well as soybean hull diet (SH) and saponins treated SH diet (SHS).

In total, 2,413,922 raw reads were obtained for the bacterial 16S rRNA genes in the four groups. After screening, 2,279,620 effective tags were obtained, accounting for 94.5% of the raw reads. The Good’s coverage value for all samples was greater than 99.5%. The results of the PCoA with unweighted UniFrac distances indicated that the four treatment groups were largely separated from each other at the OTU level (Figure 1). There were 1,339 OTUs that were identified in AH and AHS, among which 803 OTUs were found in both groups and accounted for 60.0% of the total OTUs, indicating the presence of a large common microbiome (Supplementary Figure S1). There were 925 OTUs that were identified in SH and SHS, among which 497 OTUs were found in both groups and accounted for 53.7% of the total OTUs (Supplementary Figure S1). No difference in OTU was found between AH and AHS. Compared with SH, the SHS group had more OTUs (*P* < 0.01; Supplementary Figure S1). The total number of tags that classified the microbial species after annotations for all the individual samples was greater in the AHS and SHS groups than in the AH and SH groups (Supplementary Figure S2). A similar level of species richness existed between AH and AHS based on the Chao1 index (Supplementary Figure S3). Compared to the SH group, the SHS group had a significantly greater richness (*P* < 0.01) based on the Chao1 index (Supplementary Figure S3B). The Shannon index analysis indicated that there was a similar tendency of diversity and uniformity between AH and AHS, as well as between SH and SHS (Supplementary Figure S3).

**FIGURE 2:**
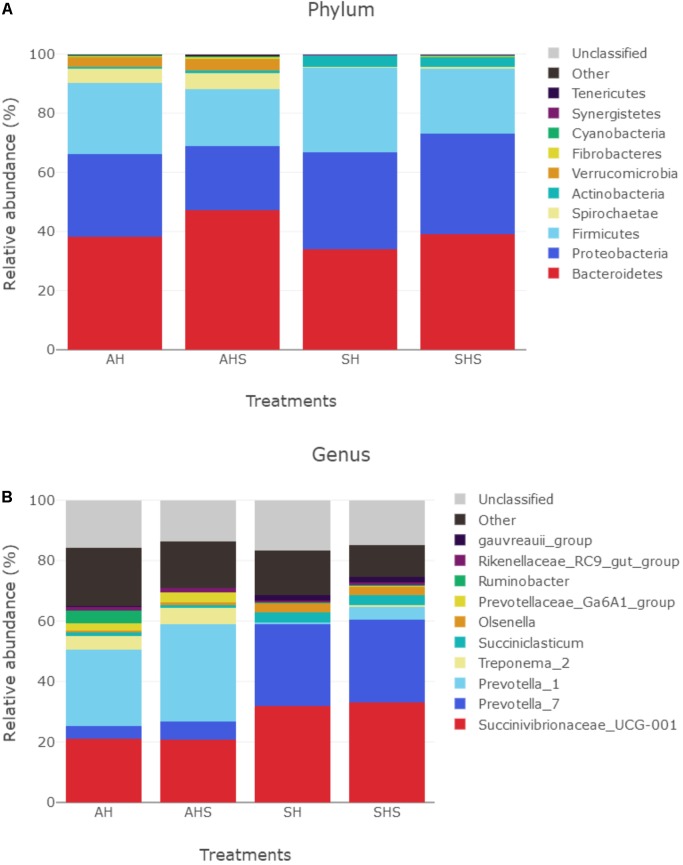
Distribution of bacterial taxa averaged under phyla **(A)** and genera **(B)** level across the dietary treatments (as a percentage of the total sequence).

In total, 17 bacterial phyla were identified in the rumen samples. Among these phyla, *Bacteroidetes*, *Proteobacteria*, and *Firmicutes* had relatively high abundances, with mean abundance levels of 39.6 ± 9.6% (mean ± standard deviation), 29.2 ± 14.2%, and 23.5 ± 6.8%, respectively (Figure 2A). The relative abundances of *Verrucomicrobia* (*P* < 0.01, *q* < 0.01), *Elusimicrobia* (*P* < 0.01, q < 0.01), *Bacteroidetes* (*P* < 0.05, *q* < 0.1), *Chloroflexi* (*P* < 0.05, *q* < 0.1), and *Cyanobacteria* (*P* < 0.05, *q* < 0.1) were greater in SHS compared to SH. However, the relative abundance of *Firmicutes* was lower in SHS compared to SH (*P* < 0.05, *q* < 0.1).

There were 330 bacterial taxa identified at the genus level, and 61 genera were present in all samples, which was indicative of the core microbiome in this study (Figure 2B). *Succinivibrionaceae_UCG-001* (26.8 ± 13.6%), *Prevotella_7* (16.0 ± 11.7%), *Prevotella_1* (15.6 ± 16.3%), *Treponema_2* (15.6 ± 16.3%), *Succiniclasticum* (2.7 ± 3.5%), *Olsenella* (1.77 ± 1.72%), *Prevotellaceae_Ga6A1* (1.48 ± 1.95%), *Ruminobacter* (1.18 ± 4.3%), *Rikenellaceae_RC9_gut* (1.05 ± 0.6%), and *gauvreauii* (1.01 ± 1.05%) were considered high-abundance taxa (Figure 2). Table 2 compares the ruminal bacteria diversity between the diets. Among these, 5 and 14 different genera bacteria were identified in comparisons between AH and AHS and between SH and SHS, respectively. The percentages of *Ruminococcaceae_NK4A214* (*P* = 0.04), *Lachnospiraceae_NK3A20* (*P* = 0.05), and *Ruminococcaceae_ UCG-008* (*P* < 0.01) were lower in AHS compared to AH (Table 2). In contrast, the proportions of *Prevotellaceae_YAB2003* (*P* < 0.01) and *Sphaerochaeta* (*P* < 0.01) were greater in AHS compared to AH. However, there was no difference at genus level between AH and AHS after FDR correction. The proportions of *Lachnospiraceae_NA* (*P* = 0.01), *Lachnospiraceae_NK3A20* (*P* = 0.02), *Erysipelotrichaceae_UCG-002* (*P* = 0.04), *Ruminococcaceae_NK4A214* (*P* < 0.01), *Syntrophococcus* (*P* < 0.01), and *Pseudoramibacter* (*P* = 0.03) decreased when the calves were fed SHS compared to those when the calves were fed SH. However, the proportions of *Prevotella_1* (*P* < 0.01), *Treponema_2* (*P* = 0.04), *Christensenellaceae_ R-7* (*P* < 0.01), *Prevotellaceae_Ga6A1* (*P* < 0.01), *Clostridium_ sensu_stricto_1* (*P* < 0.01), *Prevotellaceae_UCG-001* (*P* < 0.01), *Ruminococcaceae_UCG-002* (*P* = 0.01), and *Prevotellaceae_ YAB2003_group* (*P* < 0.01) were increased when the calves were fed SHS compared to calves fed SH. However, the significance of the differences at the genus level of *Lachnospiraceae_ NA*, *Lachnospiraceae_NK3A20*, *Erysipelotrichaceae_UCG-002*, *Pseudoramibacter*, *Prevotellaceae_UCG-001*, and *Treponema_2* between SH and SHS was lost after FDR correction.

**Table 2:** Main microbiota (accounting for ≥0.05% of the total sequences in at least one of the samples) that significantly changed between saponins treated group and control (abundance of the genera is expressed as a percentage).

	Genus	Treatments				
Phylum^a^		Control	Saponin-treated	SEM	*P*-values	FDR^b^
AH vs. AHS						
*Firmicutes*	*Ruminococcaceae_NK4A214*	1.97	0.91	0.352	0.04	0.63
	*Ruminococcaceae_UCG-008*	0.20	0.08	0.032	<0.01	0.52
	*Lachnospiraceae_NK3A20p*	0.80	0.33	0.138	0.05	0.63
*Bacteroidetes*	*Prevotellaceae_YAB2003*	0.06	0.17	0.025	<0.01	0.50
*Spirochaetae*	*Sphaerochaeta*	0.02	0.06	0.013	0.05	0.63
SH vs. SHS						
*Firmicutes*	*Lachnospiraceae_NA*	7.03	4.56	0.583	0.01	0.09
	*Lachnospiraceae_NK3A20*	2.16	0.69	0.353	0.02	0.10
	*Erysipelotrichaceae_UCG-002*	1.34	0.59	0.227	0.04	0.19
	*Ruminococcaceae_NK4A214*	0.52	0.27	0.051	<0.01	0.05
	*Ruminococcaceae_UCG-002*	0.04	0.11	0.010	<0.01	0.02
	*Syntrophococcus*	0.45	0.24	0.030	<0.01	<0.01
	*Pseudoramibacter*	0.11	0.07	0.013	0.03	0.14
	*Christensenellaceae_R-7*	0.12	0.25	0.025	<0.01	0.05
	*Clostridium_sensu_stricto_1*	0.01	0.14	0.012	<0.01	<0.01
*Bacteroidetes*	*Prevotella_1*	0.60	4.32	0.259	<0.01	<0.01
	*Prevotellaceae_Ga6A1*	0.06	0.23	0.021	<0.01	<0.01
	*Prevotellaceae_UCG-001*	0.07	0.12	0.012	0.01	0.09
	*Prevotellaceae_YAB2003*	0.003	0.07	0.003	<0.01	<0.01
*Spirochaetae*	*Treponema_2*	0.16	0.50	0.103	0.04	0.21

*^a^AH, alfalfa hay diet; AHS, saponins treated AH; SH, soybean hull diet; SHS, saponins treated SH. ^b^FDR, false discovery rate.*

### Predicted Function of the Rumen Microbiota in Response to Saponins

**FIGURE 3:**
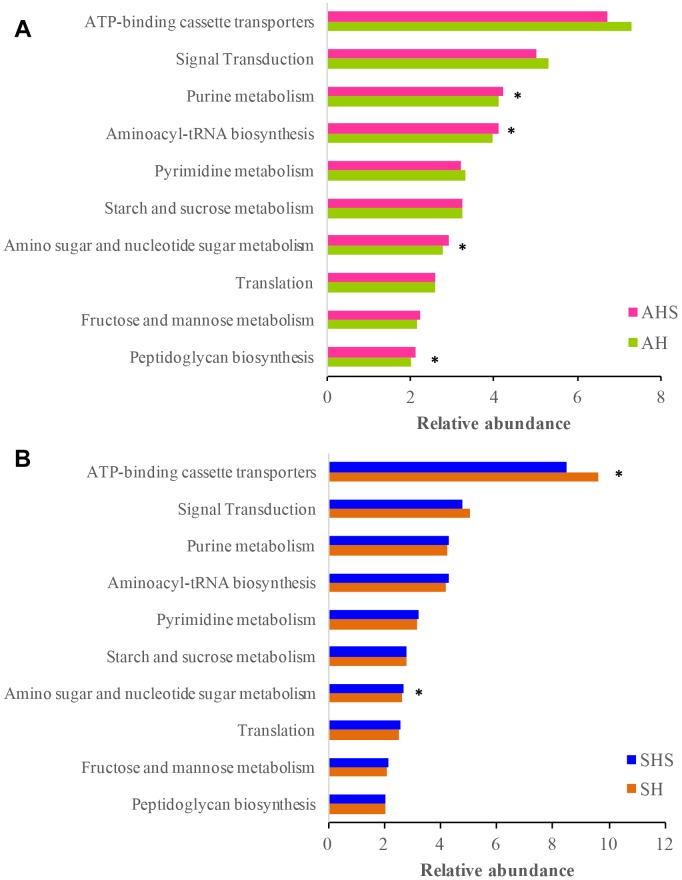
Predicted microbial functions using Tax4fun using **(A)** the differential bacteria between AH and saponins treated AHS, and **(B)** the differential bacteria between SH and SHS.

The Tax4Fun-based functional predictions revealed the 10 most important functions of the rumen microbiota (Figure 3). These functions were ‘ATP-binding cassette transporters,’ ‘signal transduction,’ ‘purine metabolism,’ ‘aminoacyl-tRNA biosynthesis,’ ‘pyrimidine metabolism,’ ‘starch and sucrose metabolism,’ ‘amino sugar and nucleotide sugar metabolism,’ ‘translation,’ ‘fructose and mannose metabolism,’ and ‘peptidoglycan biosynthesis.’ In the ruminal bacterial community, the functions related to ‘purine metabolism,’ ‘aminoacyl-tRNA biosynthesis,’ ‘amino sugar and nucleotide sugar metabolism,’ and ‘peptidoglycan biosynthesis’ were more prevalent in AHS than in AH (Figure 3A). The function of ‘ATP-binding cassette transporters’ was less prevalent in SHS than in SH, and ‘amino sugar and nucleotide sugar metabolism’ was more prevalent in SHS than in SH (Figure 3B). The mutual pathway between AH vs. AHS and SH vs. SHS was ‘amino sugar and nucleotide sugar metabolism.’

### Metabolomic Profiles in the Rumen

No obvious separation of the unsupervised PCA plot was found between AH and AHS (Supplementary Figure S4). The unsupervised PCA revealed a noticeable separation between SH and SHS (Supplementary Figure S5). Good separation of the rumen metabolites between the non-saponin and saponin groups was achieved, as shown in the OPLS-DA score plots (Supplementary Figures S4, S5). The parameters for the classifications from the software were stable and relevant to fitness and prediction (Supplementary Table S2).

### Identification of Metabolites

Both positive and negative models contained 24 samples and 5 quality control samples. Among these, 1,136 peaks were positive, and 1209 peaks were negative. In the positive model, 95 peaks were identified as differential peaks between AH and AHS, and 179 peaks were identified as differential peaks between SH and SHS. In the negative model, 19 differential peaks were found between AH and AHS, and 93 differential peaks were found between SH and SHS. In total, there were 7 DMs between AH and AHS, which were all downregulated in the calves fed AHS compared to in the calves fed AH. There were 46 DMs between SH and SHS, of which 19 DMs were downregulated and 27 DMs were upregulated in SHS compared to in SH. No mutual metabolites were identified between the DMs in AH vs. AHS and SH vs. SHS.

**Table 3:** Candidate ruminal metabolites that differed between the control and treatment.

Compounds^a^	VIP^b^	*P*-value	log2FC	*Q*-value^c^
**AH vs. AHS**				
**Positive**				
*trans*-3-Coumaric acid	1.71	0.050	−0.36	0.42
Senecioic acid	1.65	0.039	−0.53	0.40
Traumatic Acid	2.12	0.006	−0.62	0.25
Isovaleric acid	1.75	0.022	−0.64	0.35
Hypoxanthine	2.01	0.019	−0.93	0.33
Tyramine	1.79	0.011	−1.07	0.29
Pipecolic acid	1.90	0.006	−1.10	0.25
**SH vs. SHS**				
**Positive**				
Phloretin	1.78	0.041	−2.20	0.20
Daidzein	1.63	0.024	−1.62	0.18
Estradiol	1.99	0.024	−1.25	0.18
Anthranilic acid	1.77	0.011	−0.98	0.16
Glutaric acid	1.92	0.002	−0.90	0.13
Arg–Arg	1.78	0.012	−0.74	0.16
Lys-Leu	2.02	0.023	−0.71	0.18
Phenylalanine	1.51	0.026	−0.68	0.19
Arg-Glu	1.78	0.017	−0.65	0.18
5-Aminopentanoic acid	1.71	0.022	−0.56	0.18
Norvaline	1.78	0.018	−0.53	0.18
Thymidine	1.63	0.019	−0.49	0.18
Lisinopril	1.64	0.014	−0.49	0.17
Dihydrouracil	1.53	0.027	−0.40	0.19
Thiamine	1.55	0.030	−0.39	0.19
Serotonin	1.65	0.037	−0.33	0.19
His-Ala	1.78	0.016	−0.33	0.17
Tyr-Pro	1.67	0.021	−0.32	0.18
Palmitic acid	1.56	0.040	0.46	0.19
Biotin	1.54	0.042	0.63	0.20
5-Thymidylic acid	1.52	0.026	0.63	0.19
Cytidine	1.50	0.035	0.67	0.19
Thymine	1.55	0.035	0.88	0.19
Cytosine	1.74	0.004	0.96	0.13
Adenine	1.58	0.014	0.96	0.17
Phe-Ser	1.59	0.013	0.97	0.17
Deoxyadenosine	1.55	0.016	1.02	0.17
Pro-Met	1.84	0.023	1.06	0.18
Deoxycytidine	1.92	0.001	1.13	0.13
Lys-Phe	1.51	0.027	1.18	0.19
Lanosterol	2.03	0.012	1.60	0.16
Squalene	2.20	0.003	1.96	0.13
**Negative**				
Valine	1.73	0.022	−0.75	0.21
Suberic acid	1.62	0.033	0.29	0.25
2-Hydroxy-3-methylbutyric acid	1.55	0.043	0.48	0.27
Arabinose	1.55	0.038	0.53	0.26
*N*-Acetyl-L-aspartic acid	1.75	0.017	0.54	0.19
Cytidine 2′,3′-cyclic phosphate	1.61	0.030	0.59	0.24
3-Methyluridine	2.11	0.004	0.63	0.12
Nervonic acid	2.04	0.011	0.64	0.16
Glutathione disulfide	1.98	0.021	0.65	0.21
Xanthine	2.08	0.007	0.68	0.14
Pristanic acid	1.61	0.048	0.69	0.28
Citrulline	2.33	0.000	0.81	0.05
3-Phenylpropanoic acid	2.09	0.006	0.81	0.14
Deoxyguanosine	1.85	0.003	1.20	0.12
5′-Deoxyadenosine	1.80	0.016	1.39	0.18

*^a^AH, alfalfa hay diet; AHS, saponins treated AH; SH, soybean hull diet; SHS, saponins treated SH. ^b^VIP, variable importance in the projection. ^c^Q-value was used to adjust the false discovery rate in the comparisons.*

The main DMs between AHS and AH (Table 3) were hypoxanthine (log2FC = −0.93), which is a purine derivative and tyramine (log2FC = −1.07), which is a phenethylamine. The main DMs between SHS and SH (Table 3) were dihydrouracil (log_2_FC = −0.40), cytidine (log_2_FC = 0.67), deoxycytidine (log_2_FC = 1.13), 5-thymidylic acid (log_2_FC = 0.63), thymine (log_2_FC = 0.88), and thymidine (log_2_FC = −0.49), which were enriched in the pathway of pyrimidine metabolism. Among the other DMs between SHS and SH, deoxyguanosine (log_2_FC = 1.02), adenine (log_2_FC = 0.96), xanthine (log_2_FC = 0.68), and deoxyadenosine (log_2_FC = 1.02) were enriched in the pathway of purine metabolism, and squalene (log_2_FC = 1.02) and lanosterin (log_2_FC = 1.02) were enriched in the pathway of steroid biosynthesis. However, only the significance of the difference in citrulline between SH and SHS was maintained after *Q*-value correction.

**FIGURE 4:**
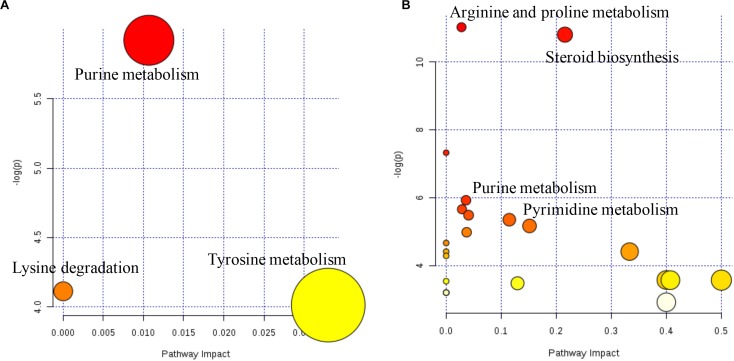
Ruminal metabolomics pathway analysis on the steers with the saponins treated compare to the non-saponins treated diet according to *Bos taurus* kyoto encyclopedia of genes and genomes pathway database alfalfa hay group **(A)**, soybean hull group **(B)**.

The metabolome map revealed the pathways based on the DMs that were identified between AH and AHS (Figure 4). The pathways of purine metabolism and tyrosine metabolism were enriched from the DMs between AH and AHS (Figure 4A). The pathways of steroid biosynthesis, pyrimidine metabolism, pantothenate and CoA biosynthesis, purine metabolism, and arginine and proline metabolism were enriched from the DMs between SH and SHS (Figure 4B).

### Correlation Between the Rumen Microbiome and Metabolome

**FIGURE 5:**
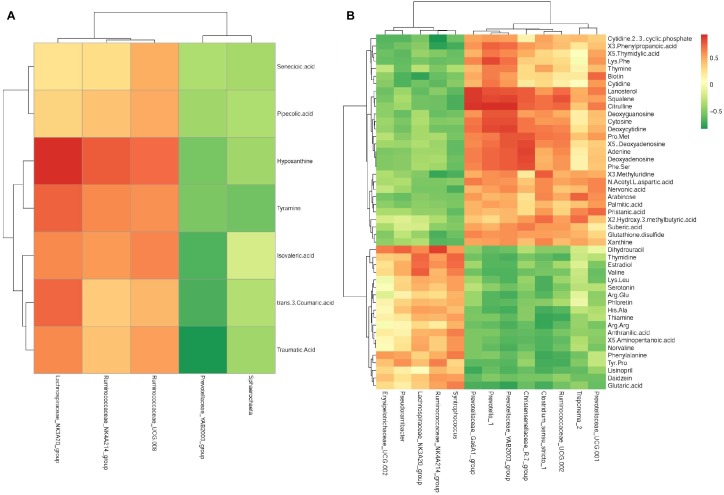
Correlation matrix between the ruminal differential metabolites (used in Table 2) affected by the saponins treatment and the differential microbiota at the genera level (used in Table 3). Positive correlations are shown in red and negative correlations in green. Color intensity is proportional to the correlation values [r] within a correlation group **(A)** alfalfa hay group, **(B)** soybean hull group.

In young growing cattle offered AH, *Ruminococcaceae_NK4A214* (*r* = 0.74, *P* < 0.01) and *Lachnospiraceae_NK3A20* (*r* = 0.90, *P* < 0.01) were positively correlated with hypoxanthine, and *Lachnospiraceae_NK3A20* was positively correlated (*r* = 0.72, *P* < 0.01) with tyramine. *Prevotellaceae_YAB2003* was negatively correlated (*r* = −0.79, *P* < 0.01) with traumatic acid (Figure 5A).

In young growing cattle offered SH (Figure 5B), *Prevotella_1* was positively correlated (*r* > 0.71, *P* < 0.01) with citrulline, squalene, deoxyguanosine, phenylpropanoic acid, Phe-Ser, Lys-Phe, lanosterol, deoxycytidine, deoxyadenosine, cytosine, and adenine. *Lachnospiraceae_NK3A20* was positively correlated (*r* > 0.73, *P* < 0.01) with estradiol and valine and negatively correlated with biotin (*r* = −0.75, *P* < 0.01). *Prevotellaceae_ YAB2003* was positively correlated (*r* > 0.71, *P* < 0.01) with citrulline, deoxyguanosine, deoxyadenosine, squalene, Pro-Met, Phe-Ser, lanosterol, deoxycytidine, cytosine, and adenine and negatively correlated with glutaric acid (*r* = −0.75, *P* < 0.01). *Treponema_2* was positively correlated (*r* > 0.71, *P* < 0.01) with 2-hydroxy-3-methylbutyric acid and arabinose. *Ruminococcaceae_NK4A214* was negatively correlated (*r* < −0.71, *P* < 0.01) with cytidine 2′,3′-cyclic phosphate, 3-phenylpropanoic acid, and 3-methyluridine and was positively correlated (*r* = 0.86, *P* < 0.01) with dihydrouracil. *Christensenellaceae_R.7_group* was positively correlated (*r* < −0.71, *P* < 0.01) with adenine, deoxyadenosine, lanosterol, Phe-Ser, Pro-Met, squalene, 5′-deoxyadenosine, and citrulline. *Clostridium_sensu_stricto_1* was positively correlated (*r* > 0.71, *P* < 0.01) with 2-hydroxy-3-methylbutyric acid and 3-methyluridine and was negatively correlated (*r* < −0.71, *P* < 0.01) with thiamine, Arg–Arg, and phenylalanine. *Prevotellaceae_Ga6A1* was positively correlated (*r* > 0.89, *P* < 0.01) with lanosterol, squalene, and citrulline. *Prevotellaceae_UCG.001* was positively correlated (*r* > 0.71, *P* < 0.01) with biotin and pristanic acid. *Ruminococcaceae_ UCG.002* was positively correlated (*r* > 0.71, *P* < 0.01) with lanosterol, squalene, and citrulline. *Syntrophococcus* was positively correlated (*r* > 0.71, *P* < 0.01) with estradiol and thymidine. *Pseudoramibacter* was positively correlated (*r* = 0.74, *P* < 0.01) with dihydrouracil.

## Discussion

**FIGURE 6:**
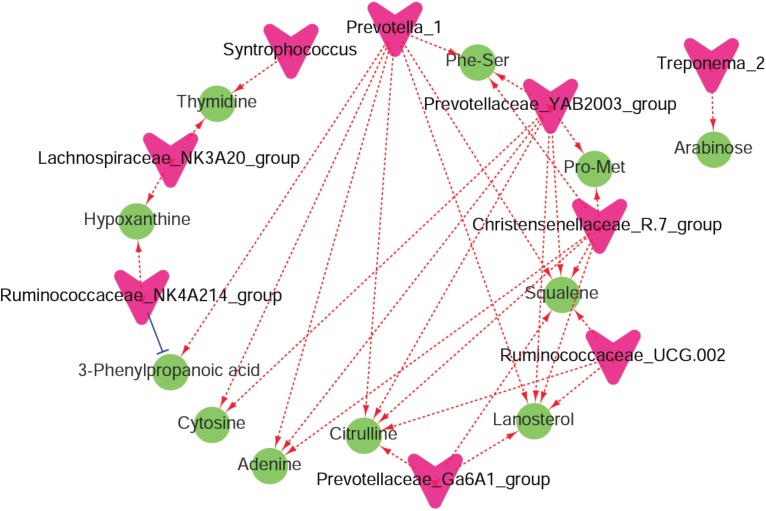
A co-occurrence network analysis among the microbiota and metabolites. Each co-occurring pair among microbial populations at the genus level and metabolites has an absolute Spearman rank correlation above 0.72 [red dotted line, positive correlation (*r* ≥ 0.72); blue dotted line, negative correlation (*r* ≤ –0.72)] with a false discovery rate-corrected significance level under 0.05. Microbes are shown by red V-shaped nodes, and metabolites are shown by green round nodes.

Recent findings showed that tea saponins have the potential to affect animal performance, modulate rumen fermentation, and might be an alternative for feed antibiotics used for growth promotion (Wang et al., 2012, 2017). Less recently, it was reported that the physiological effects of saponins appear to be less significant compared to their impact on the microbiota (Lu and Jorgensen, 1987). Similarly, in the present study, we found differences in ruminal metabolites and microbiota but similar ruminal VFA profiles in response to supplementation of tea saponins. In the current study, UHPLC/QTOF-MS and 16S rRNA analyses were conducted. This combined approach was used for the first time to elucidate mechanisms by which saponins affect rumen fermentation. The enriched differential metabolic pathways, such as ‘amino sugar and nucleotide sugar metabolism,’ that were predicted by microbial functions using Tax4Fun were similar to the enriched metabolic pathways of ‘purine metabolism’ and ‘amino acid metabolism’ using the DMs. These findings indicated that the effects of saponins on rumen metabolism mainly involve “carbohydrate metabolism,” “amino acid metabolism,” and “nucleotide metabolism.” In addition, a co-occurrence network analysis among the microbiota and metabolites that were changed by saponins supplementation was conducted to show the interactions and correlations between the rumen microbiota and metabolites (Figure 6).

The three main ruminal bacteria phyla in the current study were *Bacteroidetes*, *Proteobacteria*, and *Firmicutes*, which was similar to the results of previous studies (Rey et al., 2014; Klevenhusen et al., 2017). The higher number of *Bacteroidetes* imply that *Bacteroidetes* are more favorable for rumen development in calves than other bacteria (Smith et al., 2006). It was also reported that *Bacteroidetes* express relatively large numbers of genes encoding carbohydrate-active enzymes and therefore promote the breakdown of structural polysaccharides in the rumen (Power et al., 2014). Hemicelluloses are major components of the plant biomass and include xylans, glucomannan, and xyloglucan. Enhanced hemicellulose degradation is a good indicator of animal productivity (Mertens, 1987). Associations between bacteria belonging to *Bacteroidetes* and *Firmicutes* and the degradation of structural polysaccharides have been previously reported (Wright and Klieve, 2011; Zened et al., 2013). In a previous study, xylan supplementation increased the abundance of *Bacteroidetes* but decreased *Firmicutes* at the phylum level (Emerson and Weimer, 2017), which was similar to the results from the supplementation of saponins in the current study. In addition, hemicelluloses mainly consist of heterogeneous polysaccharides of five- and six-carbon sugars, such as xylose, arabinose, and galactose. The arabinose concentration was increased in the young growing cattle fed SHS compared to that in the young growing cattle fed SH. Therefore, the greater abundance of ruminal *Bacteroidetes* and increased level of arabinose when the calves were fed saponins indicated that hemicellulose degradation was improved.

We found that the relative abundance of *Prevotellaceae_ YAB2003* was higher but *Lachnospiraceae_NK3A20* and *Ruminococcaceae_NK4A214* were lower in the saponin-treated groups than in the untreated groups. Thus, it might indicate that these three bacteria are associated with saponins. In addition, 3-phenylpropanoic acid was highly correlated with *Prevotella_1* and *Ruminococcaceae_NK4A214*. It was reported that 3-phenylpropanoic acid improved the affinity of *Ruminococcus albus* for cellulose in continuous culture (Morrison et al., 1990). Hungate and Stack (1982) showed that 3-phenylpropanoic acid dramatically stimulated the cellulolytic activity of *Ruminococcus albus* when grown in a synthetic medium. *Ruminococcus albus* was not found in the current study, but *Ruminococcaceae_NK4A214* belongs to the same family as *Ruminococcus albus*. This finding might indicate the importance of this bacteria in fiber digestion in the rumen. However, *Treponema* was an abundant genus in cows fed an alfalfa diet, which was probably related to the degradation of pectin (Liu et al., 2015). Thus, the greater abundance of *Treponema_2* in SHS than in SH indicated that pectin degradation improved after the supplementation of saponins. In addition, arabinose was highly positively correlated with *Treponema_2*, indicating that saponins might improve the growth of ruminal *Treponema_2* and promote the release of arabinose by substrate degradation.

*Prevotella_1* and *Prevotellaceae_YAB2003* were reported to have the ability to degrade fiber sources such as hemicellulose or xylan (Emerson and Weimer, 2017). It was also reported that *Prevotella* has the capability to utilize starch, simple sugars, and other non-cellulosic polysaccharides as energy sources (Purushe et al., 2010). The polysaccharide-degrading *Prevotellaceae* were the most abundant in the rumen of cows that were fed a diet with a high starch content (Thoetkiattikul et al., 2013); however, several *Prevotella* strains are known to degrade oligosaccharides and hemicellulose (Emerson and Weimer, 2017). Thus, the greater relative abundance of *Prevotella_1* and *Prevotellaceae_YAB2003* in the saponin-treated groups (AHS and SHS) than in the untreated groups indicated that saponins effectively improve carbohydrate metabolism, especially hemicellulose digestion. In addition, *Prevotellaceae_UCG-001* and *Prevotellaceae_Ga6A1* within the *Prevotellaceae* had a greater abundance in SHS compared to SH.

A positive correlation was found between *Christensenellaceae_ R-7_group* and DMs such as citrulline, lanosterol, and squalene. It was reported that rats with a hypertriglyceridemia-related necrotizing pancreatitis had a decreased abundance of intestinal *Christensenellaceae_R-7_group* (Chen et al., 2017). Thus, the increased abundance of *Christensenellaceae_R-7_group* in the calves fed SHS might indicate the beneficial function of saponins in improving the rumen environment. Squalene belongs to the mixed triterpenoid saponin family, and tea saponins mainly contain triterpenoid saponins. Squalene is then converted to lanosterol and, after a series of reactions, is converted to cholesterol (Leon et al., 2006). The significant positive correlations between squalene and *Prevotella_1*, *Prevotellaceae_UCG-001*, and *Prevotellaceae_YAB2003_group* confirmed the importance of squalene after saponins is consumed. Lanosterol was reported to modulate TLR4-mediated innate immune responses in macrophages (Araldi et al., 2017). Thus, the increased lanosterol concentration in SHS compared to that in SH might be associated with an improved immune function in the animals after supplementation with tea saponins (Wang et al., 2017). In addition, lanosterol was highly correlated with several different bacteria at the genus level, confirming the importance of these metabolites. Citrulline is the product of arginine, and ornithine is the product of citrulline in the rumen. Ruminal microbial growth increased when ornithine was supplied to goats in peptide form (McSweeny et al., 1993). The pathway of arginine and proline metabolism was promoted in SH. The abundance of Arg–Arg was lower but citrulline was greater in SHS than in SH, indicating that saponins may have the ability to improve the degradation of Arg–Arg to citrulline by enhancing arginine catabolism.

The metabolism of saponins in the rumen involves deglycosylation (Wang et al., 2000) and structural changes of the aglycone nucleus (Wang et al., 1998). Saponins are degraded in *in vitro* rumen cultures to sapogenins (Makkar and Becker, 1997). However, the resultant sapogenins undergo structural oxidation and reduction more slowly than other saponin-related metabolites (Wang et al., 1998). There is no evidence that rumen microbes or microorganisms found in other environments are able to break down the ring structure of triterpenoid saponins (Patra and Saxena, 2009). Ingested saponins were quickly hydrolyzed in the rumen to free sapogenins and, in part, epimerized to yield episapogenins. Free sapogenin was found in the jejunum of sheep (Flåøyen et al., 2002). Thus, the efficient role of saponins in the rumen is due to the free sapogenins, and saponins might not be degraded to other metabolites to exert their functions. There was a greater concentration of daidzein in SH than in SHS, indicating that the saponins might increase the microbial utilization of daidzein, which would be beneficial to animals. Previous results suggested that supplemental daidzein can affect lipid metabolism by enhancing intramuscular fat deposition and improves meat quality in finishing steers (Zhao et al., 2015). Squalene is an intermediate metabolite in the synthesis of cholesterol. Supplementation with squalene reduced cholesterol and triglyceride levels in mice (Kelly, 1999). Thus, the supplementation of saponins might increase the intramuscular fat content and improve meat quality by affecting lipid metabolism (Kelly, 1999). In addition, it was reported that strains of the family *Lachnospiraceae* that are detected in the rumen are potentially able to biohydrogenate fatty acids (Boeckaert et al., 2009). Therefore, the relatively lower concentrations of estradiol and daidzein decreased the abundance of the *Lachnospiraceae NK3A20* in SHS, but the higher squalene concentration in SHS than in SH might help inhibit the hydrogenation of fatty acids after saponins is consumed; this role might be attributed to the antioxidant effect of tea saponins (Wang et al., 2017).

It was reported that saponins may enhance the performance of ruminants fed roughage-based diets (Patra and Saxena, 2009). Conversely, supplementing concentrate-based diets with saponins generally does not improve ruminant performance (Zinn et al., 1998). The SH-based diet could be treated as a concentrate-based diet when compared with the alfalfa hay-based diet in the present study because there was more easily fermented fiber in the SH diet than in the AH diet. A limitation of the current study is the relatively short time of saponin supplementation. In addition, the ruminal fluid collected in the current study was only from one time point (3 h after morning feeding). Thus, other time points should be included in future studies. However, we clearly found different ruminal microbiota and metabolome responses between the two diets, showing that the saponins were more effective in the SH group than in the AH group. This suggests that the effect of saponins on ruminal bacteria are diet dependent. It was proposed that this finding might be mainly due to the relatively large abundance of saponins in alfalfa, which would mask the effect of the external supplementation of tea saponins (Sen et al., 1998). Thus, studies on manipulating rumen metabolism using saponins should include different diets. However, we believe that the shortcomings of this study were mainly due to the relatively low number of rumen samples (*n* = 6), which resulted in a relatively high Q-value in the metabolome identification. Thus, future studies with more animals need to be performed to clarify the findings in this study.

## Conclusion

This study observed changes in the ruminal bacterial microbiota and metabolites in response to tea saponin supplementation. The supplementation of saponins had greater effects in SH compared to AH diets. An increased abundance of *Prevotellaceae_YAB2003 but* decreased abundances of *Ruminococcaceae_NK4A214 and Lachnospiraceae_NK3A20* were observed in young growing cattle after they were fed saponins in both of the base diets, indicating that these bacteria might be saponin-associated bacteria. Squalene, lanosterol, 3-phenylpropanoic acid, and citrulline were key metabolites detected in response to SHS. The pathways of arginine and proline metabolism, purine metabolism, and pyrimidine metabolism predicted by the DMs were affected by saponins, which was similar to the pathway of amino sugar and nucleotide sugar metabolism predicted by different microbial genes. Saponins might have the ability to change ruminal lipid metabolism in young growing cattle due to the decreased abundance of *Lachnospiraceae_NK3A20*, decreased concentrations of ruminal estradiol and daidzein, and increased squalene concentration.

## Author Contributions

BW, QD, and YT conceived and designed the experiments. BW and MM conducted the experiments and performed the statistical analysis of the experimental data. Finally, the paper was written BW, and was modified by QD and YT. All authors read and approved the final manuscript.

## Conflict of Interest Statement

The authors declare that the research was conducted in the absence of any commercial or financial relationships that could be construed as a potential conflict of interest.

## Footnotes

1 http://www.ncbi.nlm.nih.gov/Traces/sra/

2 http://drive5.com/uchime/uchime_download.html

3 http://www.drive5.com/usearch/manual/uchime_algo.html

4 https://www.arb-silva.de/

5 http://www.metaboanalyst.ca/

6 https://cran.r-project.org/package=pheatmap
