# Supplementary material for: Saponin-Induced Shifts in the Rumen Microbiome and Metabolome of Young Cattle
Source: Front Microbiol. 2019 Feb 28;10:356. doi: 10.3389/fmicb.2019.00356 (PMC6403146; doi:10.3389/fmicb.2019.00356)
Supplement: Supplementary file 1 [file Data_Sheet_1.docx]

***Supplementary Material***

**Saponin-induced Shifts in the Rumen Microbiome and Metabolome of Young Cattle**

**Bing Wang^1,2^, Man Peng Ma^1^, Qi Yu Diao^1^, Yan Tu^1^**

^1^Feed Research Institute, Chinese Academy of Agricultural Sciences, Beijing 100081, P.R. China, ^2^College of Animal Science and Technology, China Agricultural University, Beijing 100193, P. R. China

Running title: Ruminal microbial and metabolic profiles

**Correspondence:**

Dr. Yan Tu

[tuyan@caas.cn](mailto:tuyan@caas.cn)

**Table S1.** Ingredients and nutrient composition of the total mixed ration containing alfalfa hay (AH) and soybean hull (SH) as the main fiber source (DM basis)

| Items, % | AH | SH |
| --- | --- | --- |
| Ingredients |  |  |
| Corn | 41.1 | 40.5 |
| Soybean meal | 5.5 | 15.4 |
| Wheat bran | 15.0 | 15.0 |
| Fat powder | 0.40 | 0.85 |
| Alfalfa hay | 35.4 | 0 |
| Soybean hulls | 0 | 25.0 |
| Stone powder | 0.57 | 1.22 |
| Ca(HCO_3_)_2_ | 0.59 | 0.52 |
| NaCl | 0.45 | 0.43 |
| Premix^a^ | 1.0 | 1.0 |
| Nutrients |  |  |
| OM | 93.3 | 93.3 |
| CP | 15.4 | 16.2 |
| NDF | 25.6 | 26.4 |
| ADF | 13.5 | 13.6 |
| NFC | 49.3 | 47.6 |
| EE | 3.02 | 3.03 |
| ME, MJ/kg | 2.46 | 2.68 |
| Ca | 0.8 | 0.8 |
| P | 0.4 | 0.4 |

^a^Formulated to provide (per kilogram of DM): VA 15000 IU, VD 5000 IU, VE 50mg, Fe 90mg, Cu 12.5mg, mn 60mg, Zn 100mg, Se 0.3mg, I 1.0mg, Co 0.5mg.

**Table S2.** Summary of the model fitness of UHPLC-QTOF/MS analysis about the comparison between alfalfa hay diets (AH) and saponin treated AH diet (AHS), as well as the comparison between soybean hull diet (SH) and saponin treated SH diet (SHS).

| Item | Model | Type | A | N | R2X(cum) | R2Y(cum) | Q2(cum) | Title |
| --- | --- | --- | --- | --- | --- | --- | --- | --- |
| Negative | Model 06 | PCA | 3 | 12 | 0.597 |  |  | AH-AHS |
|  | Model 07 | PCA | 3 | 12 | 0.508 |  |  | SH-SHS |
|  | Model 11 | OPLS-DA | 1+1+0 | 12 | 0.24 | 0.957 | -0.411 | AH-AHS |
|  | Model 12 | OPLS-DA | 1+1+0 | 12 | 0.36 | 0.967 | 0.495 | SH-SHS |
|  |  |  |  |  |  |  |  |  |
|  |  |  |  |  |  |  |  |  |
| Positive | Model 06 | PCA | 3 | 12 | 0.548 |  |  | AH-AHS |
|  | Model 07 | PCA | 3 | 12 | 0.515 |  |  | SH-SHS |
|  | Model 11 | OPLS-DA | 1+1+0 | 12 | 0.316 | 0.963 | 0.14 | AH-AHS |
|  | Model 12 | OPLS-DA | 1+1+0 | 12 | 0.415 | 0.978 | 0.753 | SH-SHS |

*R2X and R2Y indicate the cumulative sum of squares of all the X variations and Y variations explained by all extracted components.

**Q2values display the cumulative percent of the variation in Y and can be used to estimate how well the model predicts the Y.


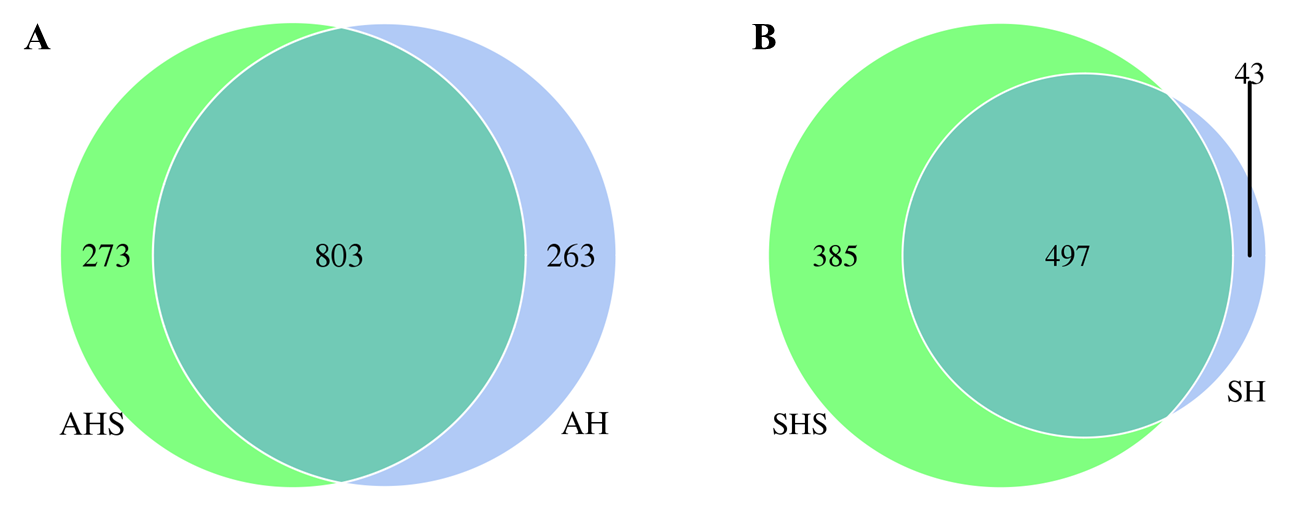


**Figure S1.** Venn diagram illustrating overlap of microbial OTUs at 3% dissimilarity level among treatments. (A) Venn diagram of bacteria between alfalfa hay diet (AH) and saponin treated AH diet (AHS); (B) Venn diagram of bacteria between soybean hull diet (SH) and saponin treated SH diet (SHS).


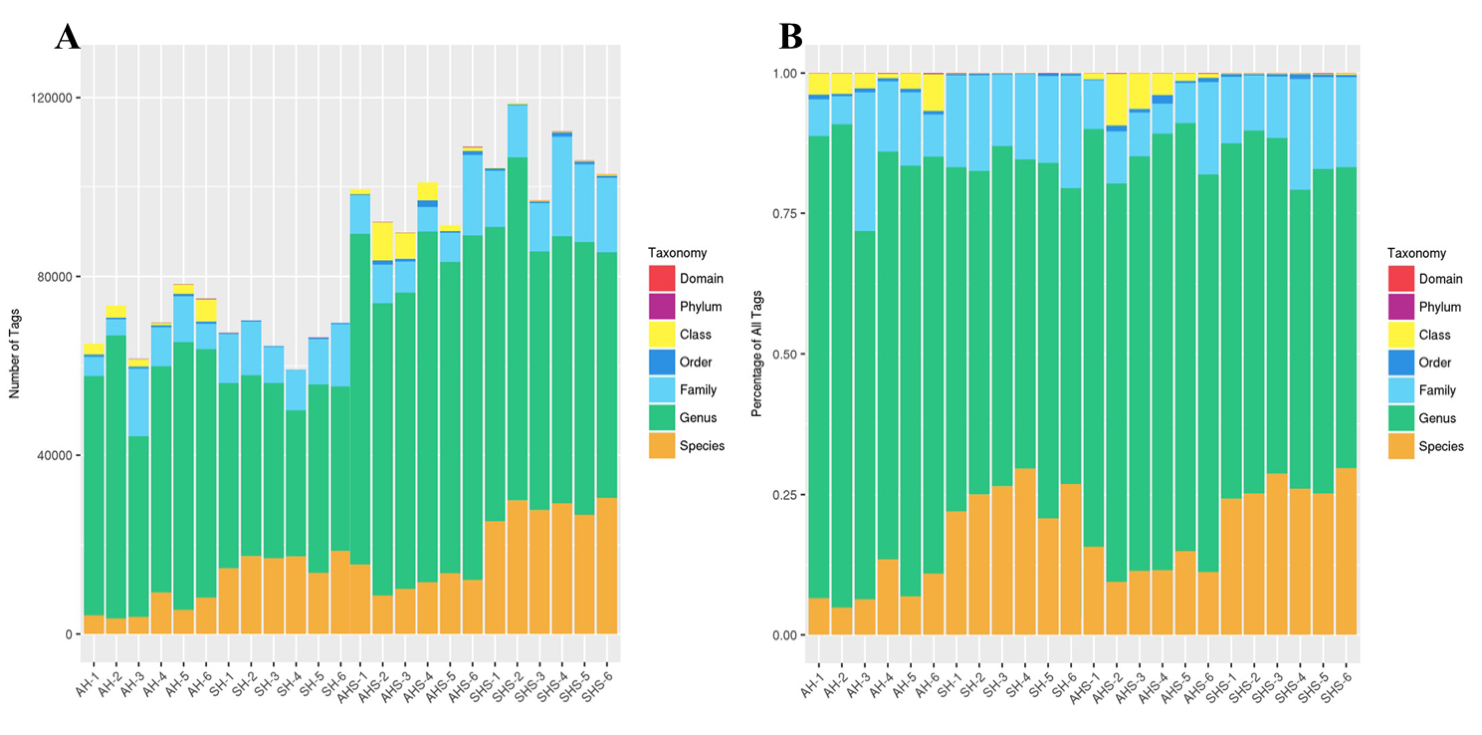


**Figure S2.** Distribution of bacterial taxonomy including domain, phylum, class, order, family, genus, species for the percentage of tags (A) and total tags (B) across all the samples.


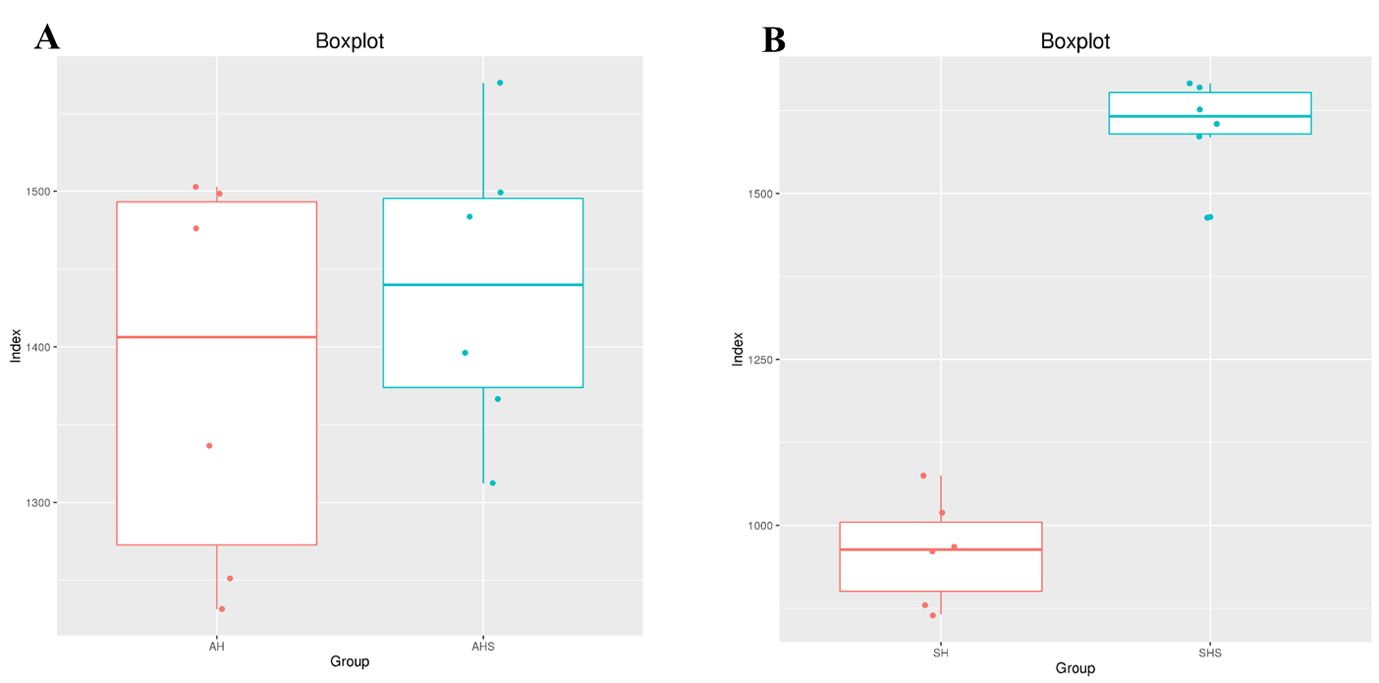
**Figure S3.** Changes in ruminal bacterial richness and diversity with supplemented of tea saponins in diets estimated by the Chao1 value (A, B) and Shannon index (C, D).


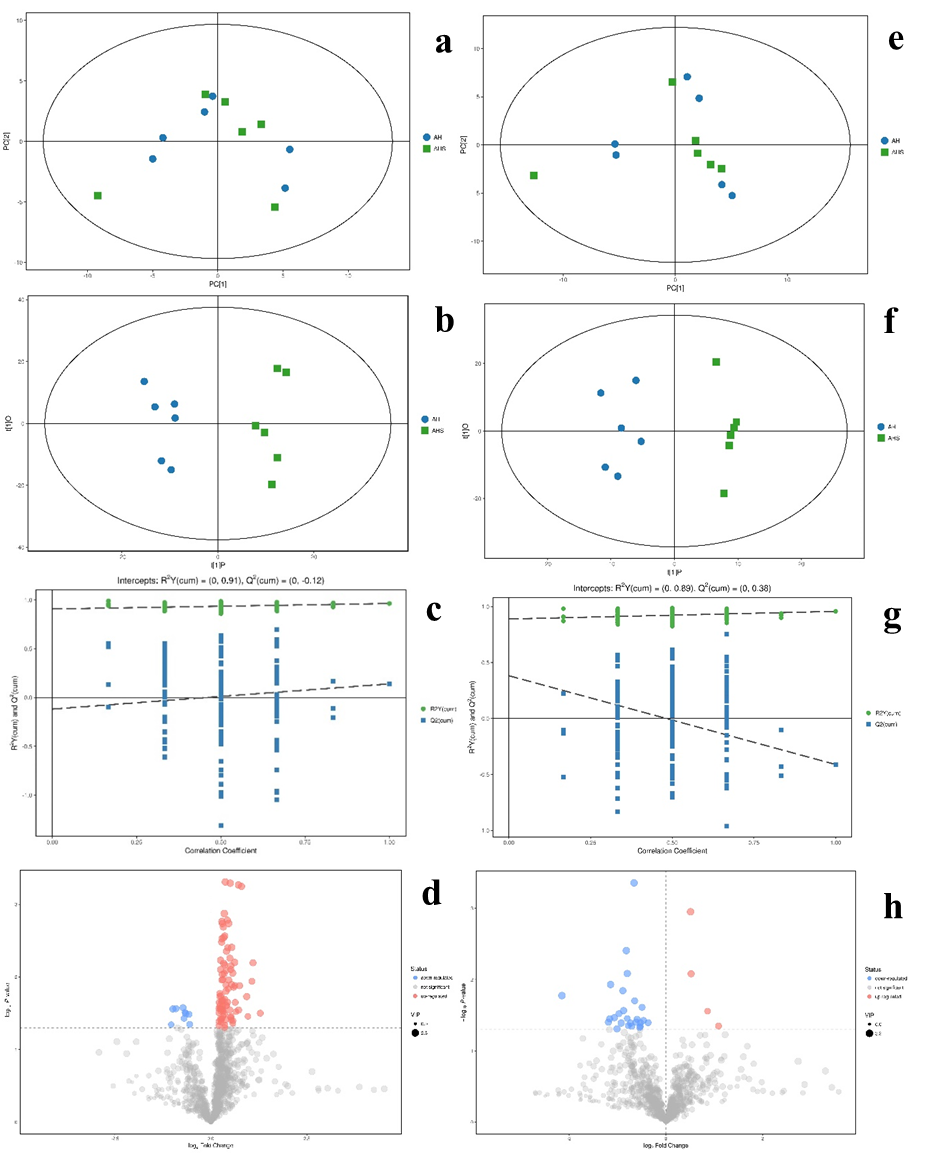


**Figure S4.** Plot of PCA (a) and OPLS-DA (b) score, and OPLS-DA permutation (c), as well as volcano plot (d) in the positive model, and plot of PCA (e) and OPLS-DA (f) score, and OPLS-DA permutation (g), as well as volcano plot (h) in the negative model, of the rumen fluid samples corresponding to the comparison between alfalfa hay diet (AH) and saponins treated AH diet (AHS).


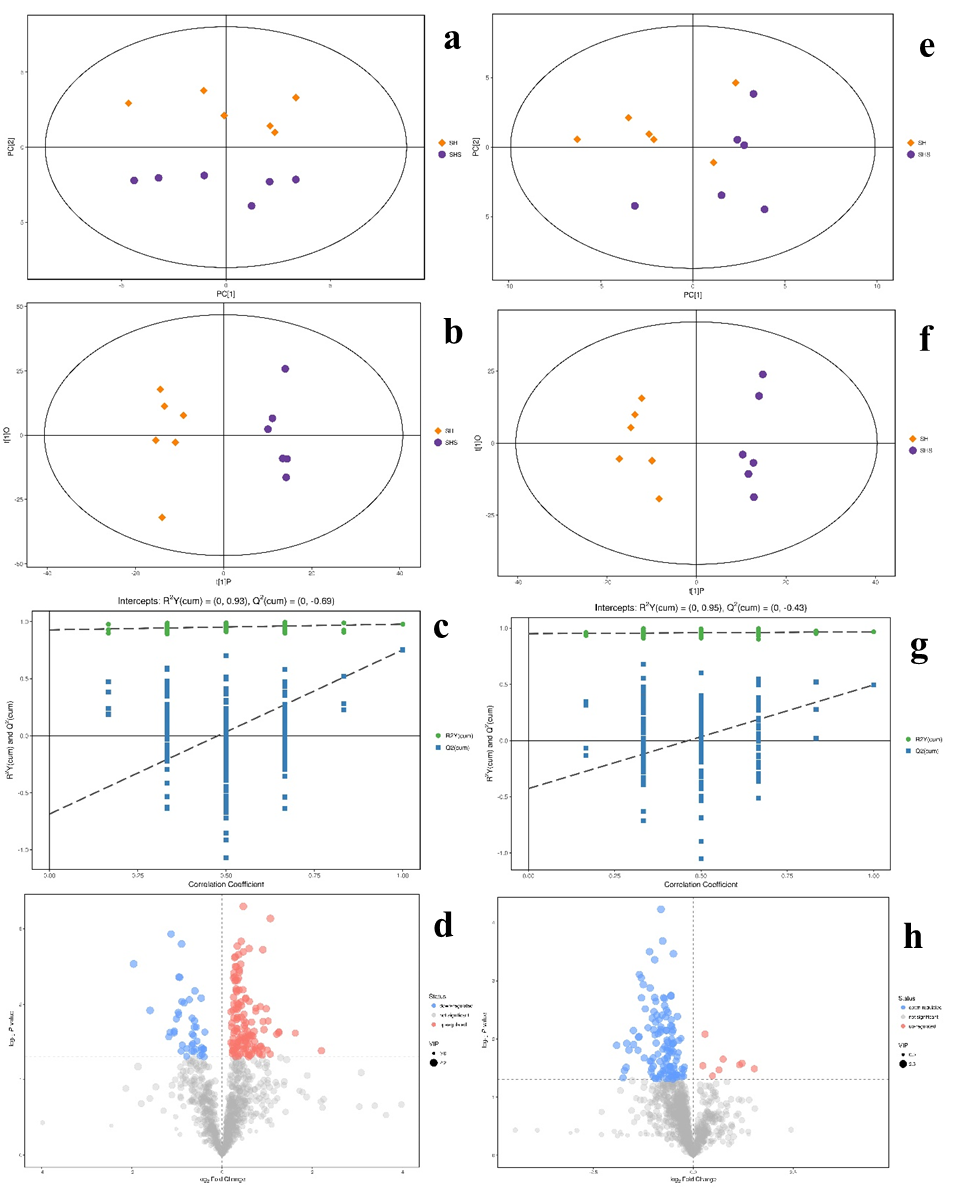
 **Figure S5.** Plot of PCA (a) and OPLS-DA (b) score, and OPLS-DA permutation (c), as well as volcano plot (d) in the positive model, and plot of PCA (e) and OPLS-DA (f) score, and OPLS-DA permutation (g), as well as volcano plot (h) in the negative model, of the rumen fluid samples corresponding to the comparison between soybean hull diet (SH) and saponins treated SH diet (SHS).
